# Editorial: Epigenetics of infectious diseases

**DOI:** 10.3389/fimmu.2022.1054151

**Published:** 2022-11-04

**Authors:** Smita Kulkarni, Thilona Arumugam, Anil Chuturgoon, Ping An, Veron Ramsuran

**Affiliations:** ^1^ Host Pathogen Interaction Program, Texas Biomedical Research Institute, San Antonio, TX, United States; ^2^ School of Laboratory Medicine and Medical Sciences, College of Health Sciences, University of KwaZulu-Natal, Durban, South Africa; ^3^ Molecular Genetic Epidemiology Section, National Laboratory for Cancer Research, National Cancer Institute at Frederick (NIH), Frederick, MD, United States; ^4^ Centre for the AIDS Programme of Research in South Africa (CAPRISA), Durban, South Africa

**Keywords:** epigenetic, methylation, infectious diseases, non-coding RNAs, histone Modifications

Host genetics contribute to variations in the acquisition, disease manifestation, and outcomes of pathogenic infections across individuals. One of the factors influencing the variability in infection and pathogenesis is epigenetics. Epigenetic factors such as DNA methylation, histone modifications, and non-coding RNAs (ncRNAs) are instrumental in host-pathogen interactions. These factors regulate pathogen and host genes without altering their genetic sequences. For instance, viruses may modify host-specific histones to make the host genome more accessible for viral replication. In contrast, the host may silence the integrated viral genome through DNA methylation, inhibiting viral replication. Thus, the series of contributions gathered in this Research Topic explores intriguing epigenetic factors that influence host-pathogen interactions. This research will drive our understanding of the role of epigenetics in immunology, pathogenesis, and possible clinical intervention of infectious diseases.

Pathogen-associated molecular patterns (PAMP) from bacteria, fungi, viruses, and protozoa may alter the epigenetic landscape of host immune cells involved in pathogen recognition. Ramendra et al. (2021) demonstrate that the potent fungal PAMP, 1,3-β- d –glucan (BDG), alters monocyte chromatin accessibility and epigenetic landscape. The changes in histone modifications in monocytes match the accessibility of the chromatin on a global scale. The changes in the epigenetic landscape prompts glutathione synthesis and metabolism, which promotes the acute functional response of monocytes to infections. The hepatitis B viral HBx protein can dysregulate host microRNA (miRNA) profiles which, in turn, modulates its viral load and enhances persistence. Sartorius et al. (2021) reviews the literature on the influence HBx has on the host and viral epigenome. The authors further attempt to link HBx dysregulated epigenetic pathways in hepatitis B virus-induced hepatocellular carcinomas.

Although our understanding of epigenetic mechanisms in infectious diseases has recently improved, its therapeutic use is still in the developmental stages. Much research has been undertaken to develop epigenetic modifying drugs for various cancers. Repurposing drugs for other diseases is an effective tool to speed up drug discovery. The histone deacetylase inhibitors (HDACi), belinostat and vorinostat, have been FDA-approved to treat various cancers. Barman et al. (2021) findings suggest that these two HDACi are effective against the *Theileria annulate* parasite in an *in vitro* model. These parasite-specific HDACi induces apoptosis in the parasite-infected cells *via* the caspase-dependent pathway while having low host cytotoxicity.

Singh et al. (2021) also repurpose an anti-cancer drug regulating DNA methylation against infectious disease, in this case, HIV. DNA methylation regulates the expression of the anti-viral restriction factor BST-2. The authors findings suggest that individuals with higher DNA methylation levels near the transcription start site of *BST2* had lower *BST2* expression and worse HIV disease outcomes. A significant negative correlation between *BST2*-methylation and *BST2* expression exists in HIV patients. Higher *BST2* expression and lower DNA methylation inhibits HIV replication in an *in vitro* HIV replication model. Treatment with a DNA-demethylating drug 5-Aza-2 –deoxycytidine increases *BST2* expression, which was associated with a lower HIV viral load.

Arumugam et al. (2021) delves further into the role of DNA methylation in the context of HIV. The authors provide a comprehensive discussion on the effect DNA methylation has on both viral and host genes. The authors also provide a detailed list of HIV-associated host genes with evidence of methylation in other disease models that should be further studied in the context of HIV. In addition, the potential use of DNA methylation as both a biomarker and therapeutic strategy against HIV is critically explored in this review.

DNA methylation not only plays a role in the pathogenesis of viral infection but also regulates the host’s immune response to bacterial infections. The changes in host methylation profiles may be brought about, in part, by the bacteria. The review by Qin et al. (2021) provides an in-depth discussion on factors regulating DNA methylation and recent insights into the regulation of host DNA methylation during bacterial infection.

NcRNAs, which include miRNAs, long non-coding RNAs (lncRNAs), and circular RNA (circRNA), are significant regulators of genes involved in the immune response. The expression of ncRNAs can differ in different physiological or disease states. Bacteria, viruses, or fungi can significantly change the pathogen and host’s ncRNA profiles in sepsis. Thus, over the past decade, more attention has been given to understanding the role of ncRNAs in disease etiology. Ghafouri-Fard et al. (2021) provide an extensive list of lncRNAs, miRNAs, and circRNAs involved in the initiation and progression of sepsis. In sepsis, these ncRNA generally interact to regulate inflammatory signaling pathways such as NF‐kB, PI3K/AKT, and JAK/STAT pathways. Sepsis often leads to multi-organ failure; however, septic cardiomyopathy may be reversible. Recent research has focused on preventing and reducing mitochondrial dysfunction, which is involved in the pathogenesis of septic cardiomyopathy. Liu and Chong (2021) summarize recent studies on the role of lncRNA in the mitochondrial dysfunction of septic cardiomyopathy.

Tamgue et al. (2021) discusses the function of ncRNAs in the etiology and control of major human tropical diseases, including tuberculosis, HIV/AIDS, and malaria, and neglected tropical diseases including leishmaniasis, African trypanosomiasis, and leprosy. The authors highlight several ncRNAs involved at different stages of these diseases. The authors describe several ncRNAs that have potential as biomarkers for disease diagnosis. They further identify and discuss knowledge gaps that warrant further investigation, such as potentially targeting ncRNAs for adjunctive therapy and vaccine development.

Exposure of the cornea to pathogens results in an inflammatory cascade, eventually leading to keratitis. Verma et al. (2021) summarize the clinical perspective of infectious keratitis, the role of epigenetics in infectious keratitis, and the potential of epigenetic modifiers in treating infectious keratitis.

Epigenetic factors may explain the heterogeneity of COVID-19 disease severity. Kgatle et al. (2021) highlight the role epigenetics play in regulating viral entry points and immunoregulatory genes during SARS-CoV-2 infection and the potential of epigenetic drug treatments against COVID-19. Roy et al. (2021) further elaborate by providing their opinion on how ncRNAs regulate macrophage plasticity during the pathogenesis of COVID-19 disease. The authors consider a pool of miRNAs and LncRNAs that regulate the expression of the SARS-CoV-2 receptor *ACE2* as potential direct targets for therapeutic manipulation. They also reason that macrophage overactivation in the lung and uncontrolled systemic inflammatory responses can be lowered by existing drugs such as the p38MAPK specific inhibitor simvastatinin and the toll-like receptor (TLR) antagonist Tocilizumab.

Evidence from animal models and *in vitro* studies suggests that chronic and severe infections alter the epigenetic landscape of immune cells, often leading to long-lasting immune suppression. Infection-induced epigenetic changes cause exhaustion, tolerance, and anergy in the immune cells making the surviving host susceptible to secondary infections. Epigenetic drugs can directly reverse drug-induced immune suppression. Abhimanyu et al. (2021) discuss studies demonstrating the reversal of infection-induced epigenetic-mediated immune suppression and postulate how these approaches could become clinically relevant to decrease post-infectious morbidity and mortality.

This Research Topic brings together contributions highlighting the importance of epigenetic processes involved in the pathogenesis of infectious diseases. Findings from novel research studies found in the Research Topic provide evidence of the dynamic interaction between the host epigenome and pathogen. Furthermore, the use of epigenome-modifying drugs are shown to be effective against pathogens in *in vitro* settings. The review articles and opinion pieces found in the Research Topic help to drive forward our understanding of the role of epigenetics in immunology, pathogenesis, and possible clinical intervention of infectious diseases. These articles also provide suggestions on what future research regarding the epigenetics of infectious diseases should hold. Thus, we hope that this Research Topic sparks new ideas in researchers who want to further explore both basic and translational aspects of epigenetic mechanisms in infectious diseases.

## Author contributions

All authors listed have made a substantial, direct, and intellectual contribution to the work and approved it for publication.

## Funding

VR was funded as a FLAIR Research Fellow (the Future Leader in African Independent Research (FLAIR) Fellowship Programme was a partnership between the African Academy of Sciences (AAS) and the Royal Society that was funded by the United Kingdom Government as part of the Global Challenge Research Fund (GCRF) (Grant No. FLAIR-FLR\R1\190204); supported by the South African Medical Research Council (SAMRC) with funds from the Department of Science and Technology (DST). Funding was also provided in part through the Sub-Saharan African Network for TB/HIV Research Excellence (SANTHE), a DELTAS Africa Initiative (Grant No. DEL-15-006) by the AAS. Support was also provided by the Grants, Innovation and Product Development unit of the South African Medical Research Council with funds received from Novartis and GSK R&D (Grant No. GSKNVS2/202101/005). SK was supported by National Institute of Allergy and Infectious Diseases: R56AI150371; R01AI157850; R21AI140956. The content is solely the responsibility of the authors and does not necessarily represent the official views of the National Institutes of Health. The authors declare that this study received funding from Novartis and GSK R&D. The funder was not involved in the study design, collection, analysis, interpretation of data, the writing of this article, or the decision to submit it for publication.

## Conflict of interest

The authors declare that the research was conducted in the absence of any commercial or financial relationships that could be construed as a potential conflict of interest.

## Publisher’s note

All claims expressed in this article are solely those of the authors and do not necessarily represent those of their affiliated organizations, or those of the publisher, the editors and the reviewers. Any product that may be evaluated in this article, or claim that may be made by its manufacturer, is not guaranteed or endorsed by the publisher.

